# Comparison of the Effects of a Bioceramic and Conventional Resin-Based Sealers on Postoperative Pain after Nonsurgical Root Canal Treatment: A Randomized Controlled Clinical Study

**DOI:** 10.3390/ma14102661

**Published:** 2021-05-19

**Authors:** Kiche Shim, Young-Eun Jang, Yemi Kim

**Affiliations:** Department of Conservative Dentistry, College of Medicine, Ewha Womans University, Seoul 07986, Korea; tlarlco@hanmail.net (K.S.); jang@ewha.ac.kr (Y.-E.J.)

**Keywords:** postoperative pain, root canal sealer, bioceramic sealer, resin-based sealer

## Abstract

Background: This clinical trial aimed to compare the effects of bioceramic sealer and resin-based sealer on the incidence and intensity of postoperative pain. Methods: Patients with anterior teeth or premolars requiring root canal treatment were assigned to group 1 (n = 51). Those with molars requiring treatment were assigned to group 2 (n = 57). In groups 1En and 2En, root canals were obturated with Endoseal MTA using the single-cone technique. In groups 1AH and 2AH, the sealer used was AH Plus with the continuous wave technique. On the day of canal filling, each patient was instructed to indicate their pain intensity over the 7 day postoperative period, at rest and, while biting, using a visual analog scale. Results: There was no significant difference in the incidence or intensity of postoperative pain between the Endoseal MTA and AH Plus groups during the 7 day postoperative period (*p* > 0.05). Less time was needed to seal the root canals with Endoseal MTA, especially in group 2 (*p* < 0.05). Conclusions: Endoseal MTA and AH Plus had similar effects on the incidence and intensity of postoperative pain. The obturation time was shorter when using Endoseal MTA compared to AH Plus.

## 1. Introduction

Pain of endodontic origin can be alleviated by root canal treatment. However, in one study, 17% of patients described the procedure as their most painful dental experience [1]. Indeed, it can be challenging for clinicians to alleviate pain during and after treatment. After root canal treatment, the incidence of postoperative pain reportedly ranges from 3% to 58% [2]. The factors associated with postoperative pain include working length determination [3], number of visits [4], gender [5], use of analgesics [6], instrumentation system [7], and root canal sealer [8]. To be more specific, extrusion of root canal sealer can disrupt periodontal tissues and cause inflammatory reactions, resulting in postoperative pain [8,9].

There are various kinds of root canal sealers; the most widely used are resin-based and bioceramic sealers, the latter of which were recently introduced. Bioceramic sealers generally contain alumina, zirconia particles, bioactive glass, calcium silicates, hydroxyapatite, and resorbable calcium phosphates [10]. These ingredients allow the sealers to resist bacterial leakage [11], make it biocompatible [12,13], and even stimulate dynamic intratubular biomineralization [14]. Furthermore, bioceramic sealers enhance endodontic treatment outcomes by facilitating the differentiation of odontoblasts [15] and the release of bioactive substances [16].

Endoseal MTA (Maruchi, Wonju, South Korea) is a premixed injectable bioceramic sealer that has garnered attention due to its convenience and positive outcomes. Endoseal MTA comprises calcium silicate, calcium aluminates and calcium sulfates, which has several advantages, such as high alkalinity, good flowability, and cytocompatibility [13,17]. In addition, it has dimensional stability during an experimental time of 30 days, while several other bioceramic sealers tend to expand [17]. In vitro and in vivo animal studies have shown that bioceramic sealers have favorable physiobiological properties [18,19,20]; however, although of great interest, there have been few clinical studies on the effects of bioceramic sealers on postoperative pain [8,21,22]. To the best of our knowledge, no clinical study has reported the effect of Endoseal MTA on post-obturation pain. Furthermore, pain is multifactorial, so it is very difficult to consider every factor potentially involved in its aggravation or diminution. Thus, a randomized controlled clinical trial is preferable to investigate postoperative pain [4,7,8,21].

The purpose of this single-blind, randomized controlled clinical trial was to compare the effects of Endoseal MTA and AH Plus on the incidence and intensity of postoperative pain.

## 2. Materials and Methods

### 2.1. Patient Allocation and Randomization

A total of 108 patients from the Department of Conservative Dentistry, Ewha Womans University Hospital, were enrolled in this study between March 2019 and May 2020. The study was approved by Ewha Womans University Medical Center (EUMC) Institutional Review Board on 18 January 2018, trial registration number of IRB no. 2018-01-021. Oral and written informed consent was obtained from all patients. In addition, all methods were performed following the approved guidelines and regulations. Patients with anterior teeth or premolars requiring root canal treatment were assigned to group 1; molars were assigned to group 2. Block randomization was performed to prevent any imbalance in the number of subjects in each group. The patients in group 1 were further randomized into group 1En (Endoseal MTA) and group 1AH (AH-Plus); those in group 2 were randomized to group 2En and group 2AH. The randomization table was managed by a study assistant, who delivered the allocated sealer to the practitioner just before obturation.

### 2.2. Inclusion and Exclusion Criteria

#### 2.2.1. Inclusion Criteria

1. Patients requiring root canal treatment who can follow treatment protocols as well as the criteria for postoperative pain evaluation;
2. Patients of age between 19 to 70 years old,

#### 2.2.2. Exclusion Criteria

1. Patients with any uncontrolled systemic diseases;
2. Patients who are pregnant;
3. Patients who refused to participate;
4. Teeth with open apex;
5. Retreatments of root canals;
6. Patients who initiated root canal treatment in other clinics.

### 2.3. Treatment Process

**Figure 1:**
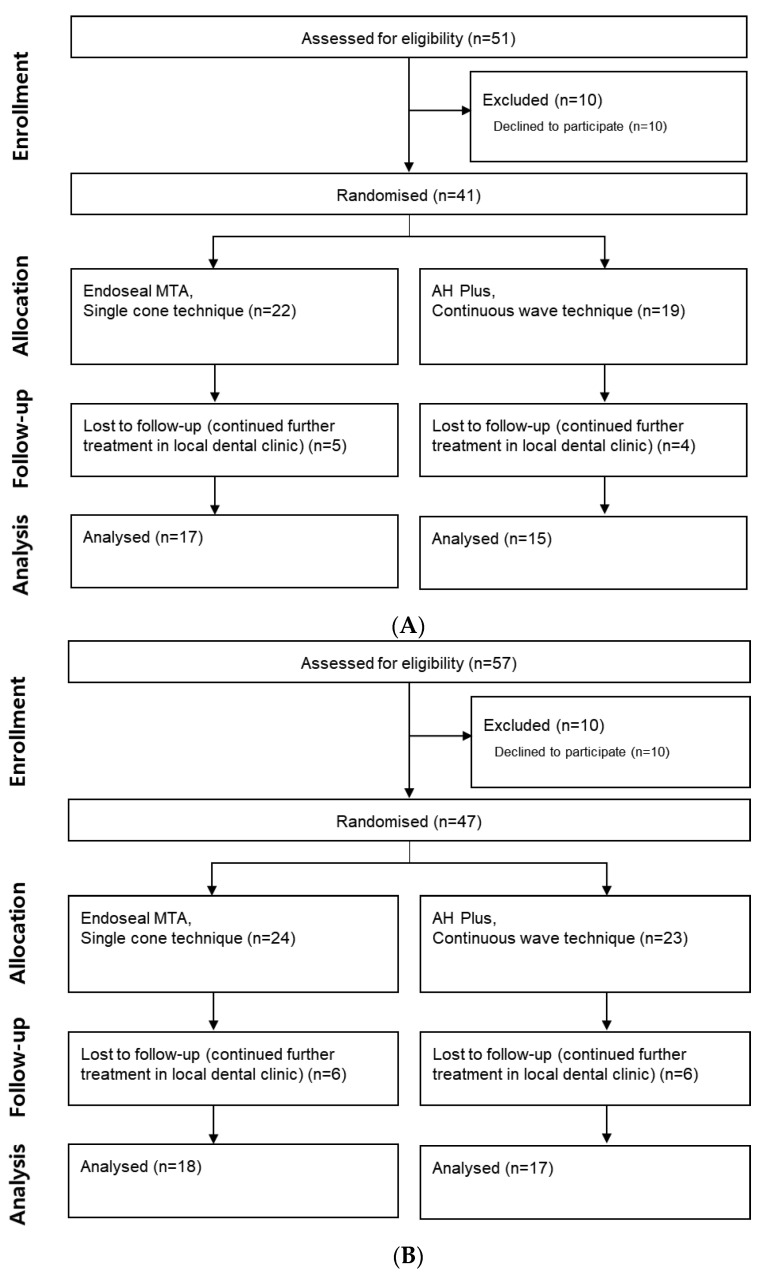
(**A**) Flow chart of patient selection in group 1. (**B**) Flowchart of patient selection in group 2.

The root canal treatment was performed by a single practitioner. Before beginning the treatment, each patient rated their preoperative pain at rest and while biting using a visual analog scale (VAS). The treatment flow diagram is provided in Figure 1A,B.

### 2.4. Root Canal Treatment Procedure

The treated tooth in all patients was anesthetized with 2% lidocaine with 1:100,000 epinephrine and isolated using a rubber dam. An access cavity was formed, and the patency of each canal was confirmed using a #15 K file. The working length was established using Root ZX (J. Morita Corp, Osaka, Japan). Each canal was shaped with the ProTaper Next file system (Dentsply Maillefer, Ballaigues, Switzerland) and soaked in 2.5% sodium hypochlorite (NaOCl) during shaping. On the day of canal filling, the canals were irrigated with 5% NaOCl for 5 min, followed by 17% EDTA for 1 min and then irrigated with 5% NaOCl and saline. Each canal was dried with paper points. An assistant delivered the assigned sealer to the practitioner just before the obturation. The continuous-wave technique was used in the AH Plus group, and the single-cone technique was used in the Endoseal MTA group. After obturation, a postoperative radiograph was taken.

### 2.5. Assessment of Postoperative Pain

On the day of obturation, each patient indicated their level of postoperative pain over 7 days, at rest and while biting, using the VAS. On day 7, each patient was asked to visit the clinic for a recall check. Their pain on that day was noted.

### 2.6. Statistical Analysis

The chi-squared test was used to compare baseline demographic characteristics between the groups. Pain incidence and intensity were analyzed by multivariate logistic regression analysis and repeated analysis of variance (ANOVA), respectively. The Mann–Whitney U test was used to analyze the obturation time data. Differences were considered significant at *p* < 0.05. SPSS software (version 25.0; SPSS Inc., Chicago, IL, USA) was used for the statistical analysis.

## 3. Results

A total of 108 patients were enrolled in this 14 month study. According to the study criteria, 20 patients were excluded, and 21 were lost to follow-up. Thus, 32 patients in group 1 and 35 in group 2 were analyzed, with a response rate of 76.13%. The average patient age was 49.04 ± 16.62 years. Table 1 shows the baseline clinical features of the patients in each group.

There were no significant differences in the effects of Endoseal MTA and AH Plus on postoperative pain (pain intensity or incidence). The postoperative VAS pain scale scores were classified as follows: no pain, 0; slight pain, 1–39; moderate pain, 40–69; and severe pain, 70–100. Most patients had no pain or slight pain. The proportions of patients with pain at rest and while biting were similar, but there was a trend toward a higher incidence of biting pain.

**Figure 2:**
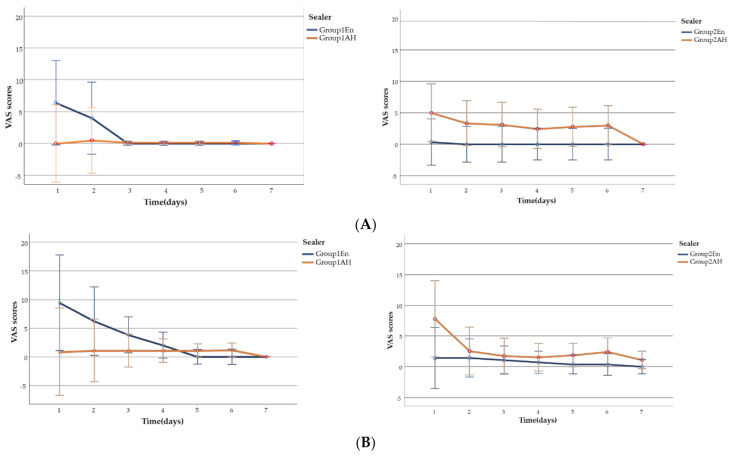
(**A**) Intensity of postoperative pain at rest over a 7 day period. The values are estimated means. The bar represents the 95% confidence interval. Repeated-measures ANOVA was used for the analysis. (**B**) Intensity of pain while biting over the 7 day postoperative period. The values are estimated means. The bar represents the 95% confidence interval. Repeated-measures ANOVA was used for the analysis.

Multiple logistic regression analysis was then conducted to determine the effects of the following potential confounding factors on pain incidence: sex, pulp vitality, and sealer. None of the preoperative factors significantly influenced the incidence of pain at any time during the 7 day postoperative period (*p* > 0.05) Repeated-measures ANOVA was conducted to determine the relationships of time and sealer with VAS pain score. The VAS scores over the 7 day postoperative period are shown in Figure 2A,B. Post-obturation pain, while biting, was analyzed separately from that and at rest. The analysis showed that: (1) VAS pain scores had a tendency to diminish over time regardless of the sealer used; (2) the VAS scores did not differ significantly over time between the sealer groups. Thus, there were no significant differences in VAS pain scores according to time or sealer.

Obturation time was compared between the sealers (Table 2). In group 1AH, more time was needed for obturation than in group 1 EN, but the difference was not statistically significant. On the other hand, group 2 EN needed 198 s for canal filling per tooth, while group 2AH needed 265 s. Differences were statistically significant (*p* = 0.004).

## 4. Discussion

Only a few studies have compared the effects of resin-based sealers, such as AH Plus, with those of other bioceramic sealers on postoperative pain; to the best of our knowledge, there have been no such studies on Endoseal MTA. Endoseal MTA is known for its high alkalinity [17] and good flowability, and cell biocompatibility [13]. Although in vitro studies have demonstrated the superior biocompatibility of Endoseal MTA over AH Plus [13], this has not been shown clinically. Bioceramic sealers are mostly used in conjunction with the single-cone technique. In contrast, AH Plus is used with the continuous wave technique. Thus, in this study, we compared the single-cone technique used in the Endoseal MTA groups with the continuous wave technique used in the AH Plus groups.

No statistically significant difference was found in pain incidence or severity between the sealers at any time point during the 7 days after obturation (*p* > 0.05). Similarly, a previous study reported no difference in postoperative pain between AH Plus (resin-based sealer) and Total Fill (bioceramic sealer), although confounding variables, such as tooth location, may have affected the results [8]. It is difficult to attribute pain to particular factors. Still, it has been suggested that the female gender is a risk factor for pain following endodontic treatment [23]. A randomized controlled design was selected for this clinical study to exclude various factors potentially associated with pain. In addition, block randomization prevented any imbalance in the number of subjects in each group. Various risk factors for postoperative pain were considered. As shown in Table 1, pulp vitality and gender showed a minor biased distribution between the groups. However, these factors did not significantly influence pain. They were adjusted for in the multivariate logistic regression analysis to ensure validity concerning the finding of no group difference in postoperative pain.

In this clinical trial, the patients were divided into anterior/premolar and molar groups because postoperative pain varies significantly by tooth type [24]. A previous study reported that pain was not experienced in 63% of anterior teeth and 44% of posterior teeth [25]. Following these studies, anterior and premolar teeth were allocated to group 1 and molars to group 2; this allowed us to exclude any effect of tooth type on pain.

Popular filling techniques, such as lateral condensation and warm vertical compaction, have drawbacks, including apical extrusion of the gutta-percha cone and insufficient gutta-percha homogeneity [26]. The single-cone technique was introduced to overcome these disadvantages. It has been proven to take less time than lateral condensation. It tightly seals the root canal without any requirement for accessory cones [26]. Compared to other obturation techniques, the single-cone technique is easier and faster [27]. Following previous studies, the obturation time in the Endoseal MTA group was shorter than that in the AH Plus group, especially in the molar group. The simplicity of this technique reduces practitioner fatigue, allowing optimal treatment to be provided to the patients [28]. One of the most important advantages of the obturation technique is apical sealing. It has been demonstrated that single-cone obturation can achieve a tight seal in the apical region [29,30]. It has also been shown that there is no significant difference in the adaptation of gutta-percha cones to root canal walls [31] or the percentage of volume voids in the apical third of root canals [32] between the continuous wave and single-cone techniques. Thus, the single-cone technique can be considered as effective as the continuous wave technique for obturation.

Bioceramic sealers are very promising but were introduced more recently than conventional resin-based sealers. Thus, further studies are required to definitively compare the clinical performance of various bioceramic sealers and postoperative pain with longer follow-ups and radiographical assessments.

## 5. Conclusions

This study showed that Endoseal MTA and AH Plus had equivalent effects on postoperative pain incidence and intensity. In addition, less time was needed for obturation in the Endoseal MTA group compared to the AH Plus group.

Thus, Endoseal MTA used in conjunction with the single-cone technique is a fast and less painful option for obturation.

## Figures and Tables

**Table 1:** Baseline clinical characteristics of the study groups. Chi-squared *p* value is presented.

Group		Group A		*p* Value	Group B		*p* Value
		Endoseal	AH Plus	-	Endoseal	AH Plus	-
Sex	Male	13	6	0.036	11	6	0.127
	Female	4	9		7	11	
						
Arch	Maxillary	9	12	0.108	5	8	0.238
	Mandibular	8	3		13	9	
						
Pulp diagnosis	Vital	10	6	0.288	7	12	0.060
	Non vital	7	9		11	5	
						
Pre-periapical lesion	Present	8	11	0.131	10	9	0.877
	Absent	9	4		8	8	
						
Pre-operative pain	Present	4	2	0.462	9	12	0.529
	Absent	13	13		6	5	
						
Sealer extrusion	Present	2	3	0.522	5	5	0.915
	Absent	15	12		13	12	

**Table 2:** Obturation time of groups 1 and 2. Mann–Whitney U test *p* value is presented.

Groups	Endoseal	AH Plus	*p* Value
Group 1, median (seconds)	129	132.5	0.396
Group 2, median (seconds)	198	265	0.004

## Data Availability

Not applicable.
